# A novel non-sense variant in the OFD1 gene caused Joubert syndrome

**DOI:** 10.3389/fgene.2022.1064762

**Published:** 2023-01-10

**Authors:** Chen Li, Xingwang Wang, Fake Li, Hongke Ding, Ling Liu, Ying Xiong, Chaoxiang Yang, Yan Zhang, Jing Wu, Aihua Yin

**Affiliations:** ^1^ Medical Genetic Center, Guangdong Women and Children Hospital, Guangzhou, China; ^2^ Medical Imaging Department, Guangdong Women and Children Hospital, Guangzhou, China

**Keywords:** Joubert syndrome, *OFD1*, novel non-sense variant, MRI, whole exome sequencing (WES), RNA-seq

## Abstract

**Background:** Joubert syndrome (JBS) is a rare neurodevelopmental disorder associated with progressive renal, liver, and retinal involvement that exhibits heterogeneity in both clinical manifestations and genetic etiology. Therefore, it is difficult to make a definite prenatal diagnosis.

**Methods:** Whole-exome sequencing and Sanger sequencing were performed to screen the causative gene variants in a suspected JBS family. RNA-seq and protein model prediction were performed to clarify the potential pathogenic mechanism. A more comprehensive review of previously reported cases with *OFD1* variants is presented and may help to establish a genotype–phenotype.

**Results:** We identified a novel non-sense variant in the *OFD1* gene, *OFD1* (NM_003611.3): c.2848A>T (p.Lys950Ter). Sanger sequencing confirmed cosegregation among this family. RNA-seq confirmed that partial degradation of mutant transcripts, which was predicted to be caused by the non-sense-mediated mRNA decay (NMD) mechanism, may explain the reduction in the proportion of mutant transcripts. Protein structure prediction of the non-sense variant transcript revealed that this variant may lead to a change in the *OFD1* protein structure.

**Conclusion:** The genetic variation spectrum of JBS10 caused by *OFD1* was broadened. The novel variants further deepened our insight into the molecular mechanism of the disease.

## Introduction

Joubert syndrome (JBS) (MIM213300) is a rare neurodevelopmental disorder that is characterized by distinctive mid-hindbrain malformation, cerebellar ataxia, oculomotor apraxia, breathing difficulties, intellectual impairment, and varying degrees of developmental delay. The presence of a specific cerebellar and brainstem malformation known as the “molar tooth sign (MTS)” (Romani et al., 2013; Poretti et al., 2014) on brain magnetic resonance imaging (MRI) is one of the main diagnostic characteristics of JBS. The MTS comprises a deep interpeduncular fossa; prominent, straight, and thickened superior cerebellar peduncles; and hypoplasia of the cerebellar vermis (Maria et al., 1999). Apart from neurological involvement, JBS is also associated with gradual damage to the retina, kidneys, and liver. In addition, polydactyly has also been observed in some patients (Parisi 2019).

JBS is a clinically and genetically heterogeneous ciliated disease. To date, at least 35 subtypes of JBS have been classified according to different causative genes. Most of them are autosomal recessive, while only JBS10 (MIM 300804) is an X-linked recessive (XLR) disorder. In JBS10, hemizygous male patients are affected, and heterozygous females are asymptomatic (Coene et al., 2009).

JBS10 is caused by *OFD1* mutations. The *OFD1* protein is essential for the formation of primary cilia and the establishment of left–right asymmetry. Moreover, it has been suggested that this multitasking protein has additional functions, such as controlling cell cycle progression (Alfieri et al., 2020), participating in chromatin remodeling and DNA repair (Abramowicz et al., 2017), acting as a novel autophagy receptor that is independent from the mTOR pathway (Franco and Morleo 2021), interacting with the translation machinery and modulating the translation of specific mRNA targets in the kidney (Iaconis et al., 2017). Pleiotropy is a common characteristic of ciliopathies (Giorgio et al., 2007). Mutations in the *OFD1* gene with different degrees of severity may cause oro-facio-digital-syndrome 1 (OFD1 syndrome, MIM 311200) (Macca and Franco 2009), Joubert syndrome (JBS10), retinitis pigmentosa (RP23, MIM 300424) (Webb et al., 2012) and primary ciliary dyskinesia (PCD, MIM 244400) (Coene et al., 2009; Bukowy-Bieryllo et al., 2019). Since the first JBTS10 case was reported in 2009 (Coene et al., 2009), only 23 families (26 patients) with male patients have been reported for JBTS10, and there were a few fetal diagnoses. Thus, it remains difficult to obtain a comprehensive understanding of this rare disease.

In this study, we describe a non-consanguineous healthy Chinese couple with two fetuses who both had unexplained hypoplasia of the cerebellar vermis and polydactyly. Employing whole-exome sequencing (WES) technology, we identified a novel variant in the *OFD1* gene. Bioinformatic validations were performed to confirm the pathogenicity of the variant at the transcription and protein levels. Furthermore, we summarized a more comprehensive review of previously reported JBS10 cases with *OFD1* variants, which may help to understand how the phenotype emerges from the genotype.

## Materials and methods

### Ethical approval

The present study was approved by the institutional review board and clinical research ethics committee of Guangdong Women and Children Hospital. Informed consent was provided by all the participants or their statutory guardian in this study.

### Subjects

**FIGURE 1:**
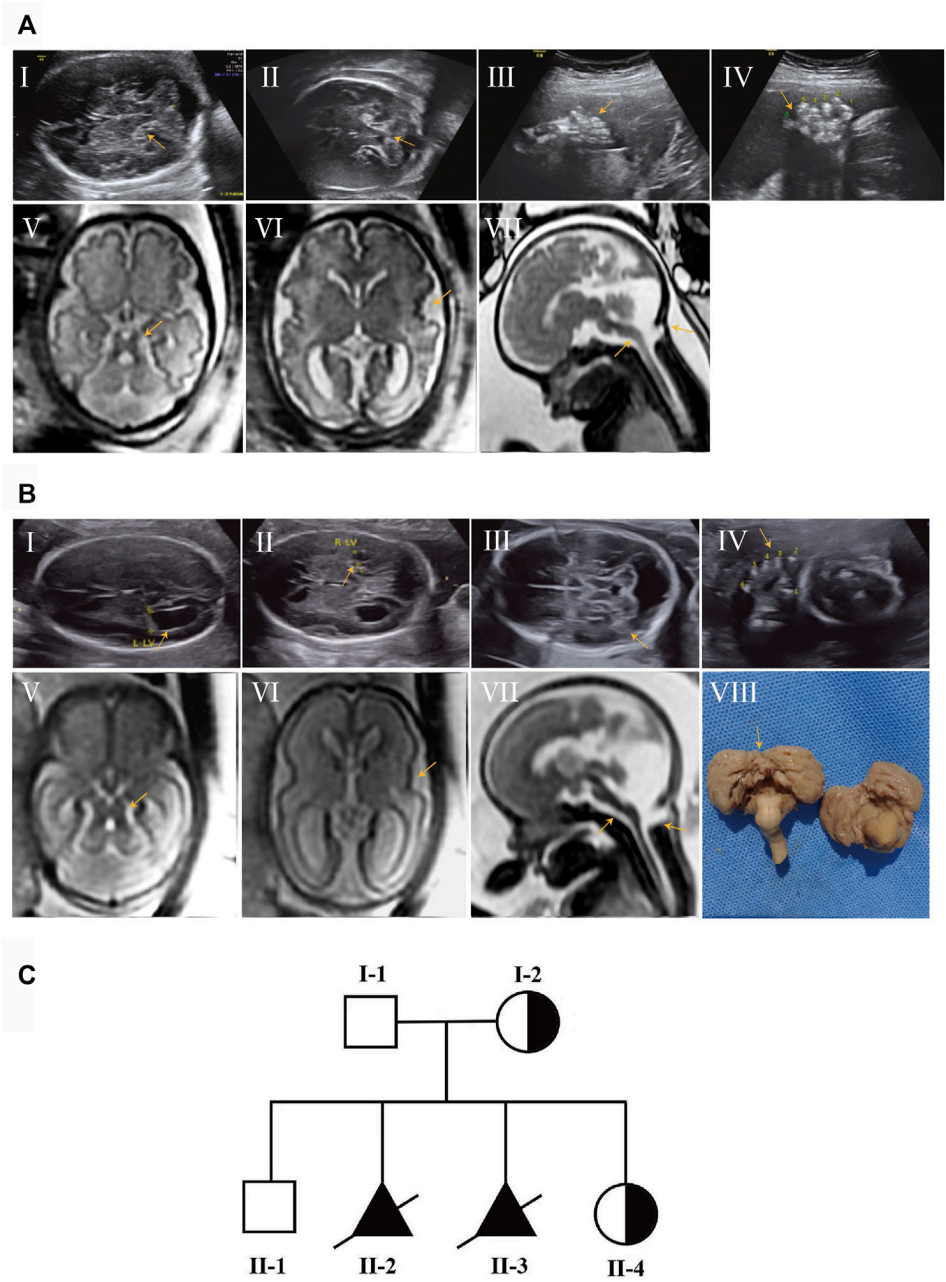
Prenatal ultrasound and fetal brain MRI findings of affected subjects and pedigree chart of the family with JBTS10. **(AⅠ–Ⅳ)** Prenatal ultrasound of II-2 showed thickening of the superior cerebellar feet, cerebellar vermis agenesis, enlarged posterior cranial fossa **(AⅠ,Ⅱ)** and bilateral postaxial polydactyly on the hands and right foot **(AⅢ,Ⅳ)** at 30 weeks of gestation. **(AⅤ–Ⅶ)** Brain MRI of II-2 showed MTS **(AⅤ)**, polymicrogyria **(AⅥ)**, posterior meningocele and thin brain stem **(AⅦ)**. **(BⅠ–Ⅳ)** Prenatal ultrasound of II-3 showed a similar anomaly to II-2 at 25 weeks of gestation. **(BⅤ–Ⅶ)** Brain MRI of II-3; polymicrogyria was not obvious compared with II-2. **(BⅧ)** Autopsy result of II-3 showed absent cerebellar vermis. **(C)** Pedigrees of the family with JBS10 profiled in this study.

A 28-year-old mother at 30 weeks of gestation was transferred to our Prenatal Diagnosis Center due to brain abnormalities and polydactyly in the fetus. Prenatal ultrasound (US) in our hospital showed thickening of the superior cerebellar feet, cerebellar vermis agenesis, enlarged posterior cranial fossa and bilateral postaxial polydactyly on the hands and right foot (Figures 1AⅠ–Ⅳ). Fetal brain MRI further displayed typical “MTS”, posterior meningocele involving the inferior portion of the occipital bone and superior cervical vertebrae, thin brain stem and polymicrogyria (Figures 1AⅤ–Ⅶ), which signified a poor prognosis. Therefore, the parents decided to terminate the pregnancy due to the fetal deformity. Umbilical cord blood sampling was performed on the fetus, which revealed normal chromosomal microarray (CMA) results. Later, trio-WES identified a novel *OFD1* variant (XLR) inherited from the mother.

This was the second child of this healthy, unrelated Chinese couple. They already had a healthy son without this *OFD1* variant. After 1 year, the mother came to us at 25 weeks of gestation for a similar abnormality (Figures 1BⅠ–Ⅶ) in the fetus. Gene detection confirmed the same *OFD1* variant in this fetus (II-3). The couple chose induced abortion and agreed to undergo further autopsy (Figure 1BⅧ). When the mother conceived for the fourth time, she came to us in the first trimester. Through chorionic villus sampling at the 13th week of gestation and normal prenatal US and brain MRI, they finally had a girl who was a carrier (Figure 1C) (Table 1).

**TABLE 1:** Clinical data of three fetuses in this family.

Subjects	II-2 (proband)	II-3	II-4
Sex	M	M	F
Gestation (weeks)	30	25	
Array CGH	NA	+(vus)	NA
OFD1 variation detection	Hemizygous	Hemizygous	Heterozygous
MTS	+	+	NA
Thin brainstem	+	+	NA
Polymicrogyria	+	+	NA
Meningocele	+	+	NA
Polydactyly	+	+	NA
Termination of pregnancy	+	+	NA

M, male; F, female; NA, not available; vus, variation of unknown significance.

### Umbilical cord blood sampling, chorionic villus sampling and DNA extraction

Fetal samples of the proband (II-2) were collected by umbilical cord blood sampling. The procedure was performed using the transabdominal approach. Under aseptic conditions, a 22-gauge spinal needle was inserted into the umbilical artery under continuous ultrasound guidance, and umbilical cord blood was collected in an EDTA vacuum tube. Samples of the fetuses (II-3, II-4) were collected by amniocentesis. The procedure was performed in much the same way as umbilical cord blood sampling, except that the spinal needle was 18–20 gauge and inserted into the placenta to obtain chorionic villus samples.

Genomic DNA was extracted from the umbilical cord blood samples (II-2, II-3), chorionic villus samples (II-4), and peripheral blood samples of the parents (I-1, I-2) and their unaffected son (II-1) by using the QIAamp DNA Mini Kit (Qiagen, Germany).

### Microarray analysis

Microarray analysis was performed using a high-resolution genotyping single nucleotide polymorphism microarray, Affymetrix CytoScan 750 K Array (Affymetrix, Santa Clara, CA, United States). Copy number variations (CNVs) were identified based on associated records of the human reference genome 37 (NCBI37hg19) of the National Center for Biotechnology Information. Data were analyzed in accordance with American College of Medical Genetics guidelines.

### Whole-exome sequencing (WES) and sanger sequencing

Briefly, the sequencing library preparation and target enrichment experiments were performed following the manufacturer’s procedures for the SureSelectXT Clinical Research Exome kit (Agilent Technologies, Santa Clara, CA). Then, paired-end reads were sequenced on a NextSeq 500 platform (Illumina, San Diego, CA) with 150-bp paired-end reads, yielding an average coverage above 110×, with 97.6% of target bases covering at least 10×. Sequence quality analysis and filtering of mapped target sequences were conducted under the “varbank” exome and genome analysis pipeline v.2.1 (Vetro et al., 2020). Standards and guidelines recommended by the American College of Medical Genetics and Genomics and the Association for Molecular Pathology were used to interpret the sequence variants (Richards et al., 2015). The genomic variation database (http://dgv.tcag.ca/dgv/app/home), DECIPHER database (https://decipher.sanger.ac.uk/), and OMIM database (http://www.ncbi.nlm.nih.gov/omim) were employed. Furthermore, the allele frequency and pLI score (representing the tolerance for truncating variants) from gnomAD databases (http://gnomad.broadinstitute.org), REVEL score (a combined method of pathogenicity prediction) (Ioannidis et al., 2016) and searching egnine for variants (Mastermind, https://mastermind.genomenon.com/) were also referenced to assist in the interpretation of variant pathogenicity. VarSome (https://varsome.com/), a widely used online pathogenicity grading tool, was employed to assess the pathogenicity of variation sites (Supplementary Figure S1).

Sanger sequencing was performed to validate the findings of trio WES in the fetuses and parents. The PCR primers for sequencing (targeting *OFD1* (NM_003611.3) c.2848A>T) were as follows: forward 5′-CAG​GCT​GTA​CTT​GAA​GGA​T-3′, reverse 5′-GTC​CCA​AGA​AGT​TAA​GCC​A-3′.

### RNA sequencing and data analysis

RNA sequencing was performed by Aegicare, which has been described in our previous literature (Qi et al., 2022). Total RNA was extracted from peripheral blood samples of one of the carriers (I-2) and two normal females using a Qiagen blood RNA extraction kit I (QIAGEN, United States) according to the manual. After complete property control and quality control of RNA, 1 μg of RNA was aspirated for mRNA library construction by using the TIANSeq Fast RNA Library Kit (Illumina, United States). Finally, the library was amplified by using a high-fidelity enzyme. After quality control, the libraries were sequenced on an Illumina HiSeq 4,000 platform.

The raw reads were subjected to quality control before mapping to the reference genome through the fastp program. Adapter sequences and low-quality reads were filtered out, and high-quality reads were mapped onto the reference genome (*Homo sapiens,* hg19) by using STAR (Dobin et al. 2013), ultrafast universal RNA-seq aligner STAR_2.3.059 with default parameters. Finally, the read count tables were generated by HTSeq (Anders et al., 2015) software, while the fragment per kilobase of transcript per million reads (FPKM) of each gene was calculated based on the length of the gene and read count tables.

The expression ratio of the *OFD1* biallelic gene is estimated by the reads containing specific SNP sites.

### Conservational and protein structural analysis

The evolutionary conservation of the affected amino acid (AA) residue by the non-sense variant was analyzed using the MEGA7 online tool (http://www.megasoftware.net/previousVersions.php) with default parameters. Protein structure was predicted by AlphaFold. RoseTTAFold online software (https://robetta.bakerlab.org/) was also used to predict the protein structure (Supplementary Figure S1).

## Results

### Chromosomal microarray (CMA) analysis

CMA was normal in the proband (II-2) but revealed a 696 kb heterozygous deletion at 5p13.33 (2091630-2787829) in the fetus (II-3), which was inherited from his mother. No similar deletion has been reported to cause disease, nor was a similar deletion found in the DGV database of the general population. The deleted region encompasses four genes. The four genes are LOC100506858, LSINCT5, IRX2, and C5orf38. LOC100506858 is a pseudogene. Although the last three genes are OMIM genes, there is no OMIM causative gene among them. Therefore, it is identified as a variants of unknown significance (VUS) according to the CNV classification guidelines of ACMG.

### Gene findings

**FIGURE 2:**
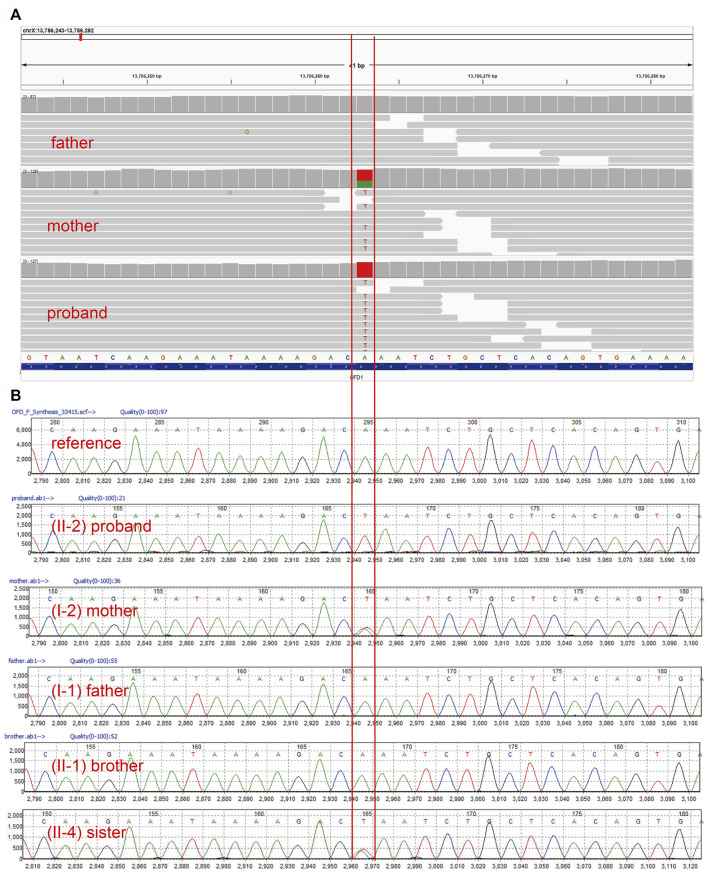
Genetic findings. **(A)**. WES detected a hemizygous variant c.2848A>T (p.Lys950Ter) of the *OFD1* gene in the index fetus, which was heterozygous in the mother and absent in the father. **(B)**. Sanger DNA sequencing chromatogram showed the cosegregation of this variant in all family members.

Trio WES identified a hemizygous variant c.2848A>T (p.Lys950Ter) in the *OFD1* (NM_003611.3) gene in the proband (II-2). The *OFD1* gene is located in the area between the pseudoautosomal boundary and the Duchenne muscular dystrophy (DMD) gene on the short arm of chromosome X. *OFD1* encodes a component of the centrioles controlling mother and daughter centriole length (Singla et al., 2010). The variant c.2848A>T (p.Lys950Ter) resulted in truncation after the first 950 residues. This non-sense mutation was absent in the population databases (GnomAD v2.1.1) and is predicted to undergo non-sense-mediated mRNA decay (NMD). The variant of the proband (II-2) was inherited from the mother, who was a heterozygous carrier. The variant cosegregated with the disease in the family, as shown by direct Sanger sequencing in all family members (Figure 2). This variant has not been reported in public databases, and few papers have examined its impact on the expression or activity of the OFD1 protein. In silico tools predicted that this variant was disease causing (Supplementary Figure S2). Taking all of these findings into consideration, this mutation can be classified as “likely pathogenic” because of the evidence chain (PVS1 + PM2) based on the ACMG guidelines (Richards et al., 2015) (Supplementary Table S1).

### Evaluation of the effect of the gene mutation on gene function

**FIGURE 3:**
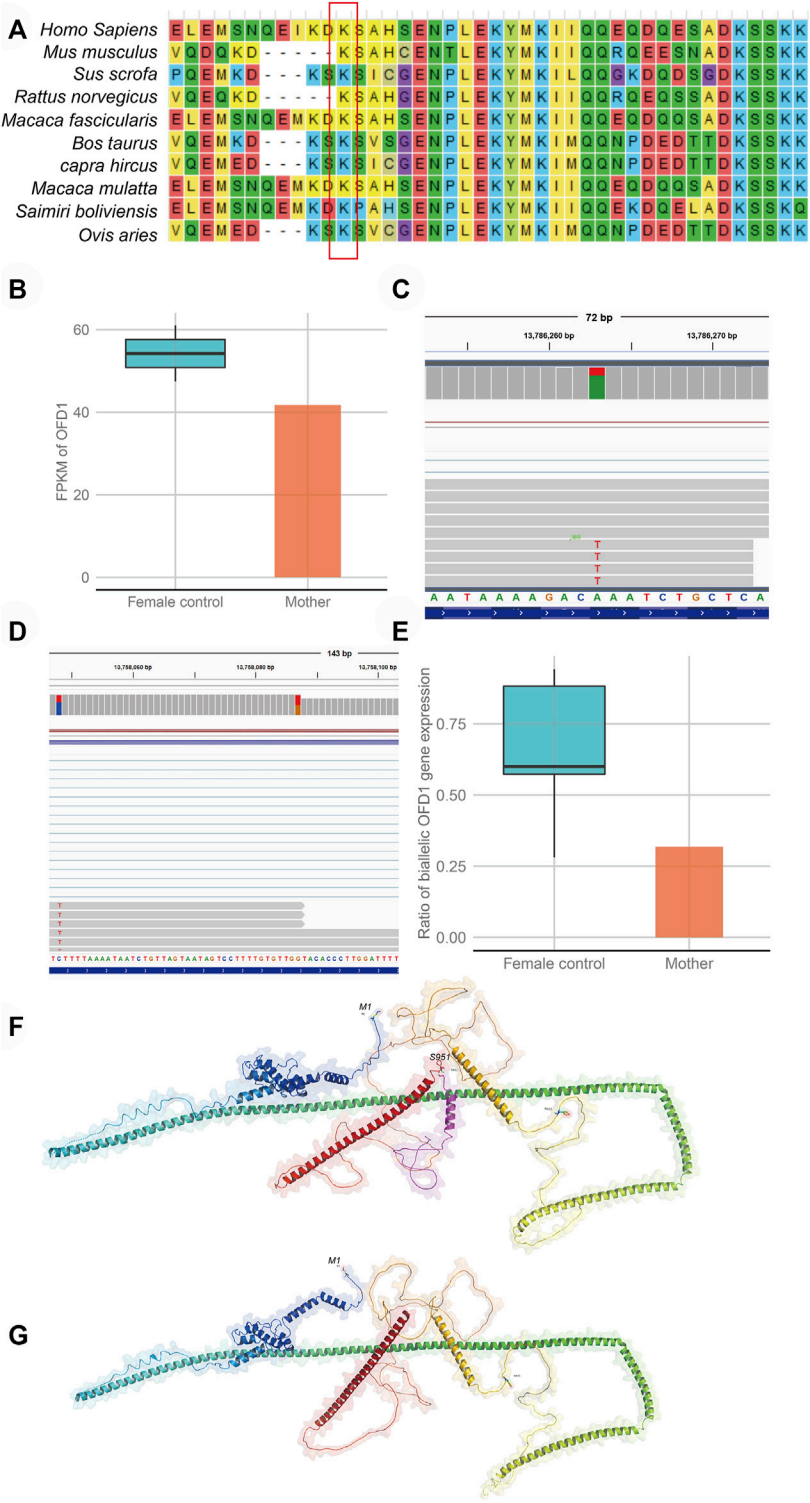
Evaluation of the effect of the gene variation on gene function **(A)**. Alignment of multiple OFD1 protein sequences across species. Letters in the red rectangle show that the regions of the variant p.Lys950Ter were highly conserved across different species. **(B)** Comparison of OFD1 expression in blood between a heterozygous mutant individual (mother) and wild-type female individuals (female controls). **(C)** Biallelic expression bias of OFD1 occurs in the mother. **(D)** Biallelic expression bias of OFD1 occurs in wild-type female individuals (female control). **(E)** The expression bias is more serious in the mutant individual (mother). **(F,G)** The predicted structure of the mutant protein and wild-type protein by AlphaFold software. **(F)** (wild-type protein), the amino acid in the red box is missing from the mutant protein. **(G)** (mutant protein).

To further investigate the influence of this variant on gene function, we performed RNA-seq on the mother’s blood sample to assess the effect of the variation on gene expression or transcript length. The results showed that *OFD1* had biallelic gene expression in all samples, which was consistent with a previous study showing that *OFD1* escaped inactivation (Figures 3C,D). Further analysis found that when the expression of *OFD1* was standardized to FPKM, its expression in the mother’s sample was lower than that in the female control samples (Figure 3B). Subsequently, we used the reads containing specific SNPs to calculate the ratio (mutant-type reads/wild-type reads) of *OFD1* biallelic gene expression and found that the ratio in the mother’s sample was lower than that in the control female sample (mutant reads/wild-type reads) (Figure 3E). This suggests that the lack of mRNA in the mother sample is due to the lack of mRNA of the mutant allele, which may be caused by RNA degradation caused by non-sense mutations. However, there was still a certain amount of mutant transcripts. This suggests that these RNAs may escape this mechanism and may be translated into proteins. The function of proteins is closely related to their structure. Therefore, we used the AlphaFold model to predict the protein spatial structure of the wild type and mutant type and found that the protein structure of the mutant type changed greatly (Figures 3F,G), which may have affected its function.

## Discussion

Herein, we report a non-consanguineous healthy Chinese couple with two male fetuses, both of whom were suspected to suffer from unexplained hypoplasia of the cerebellar vermis, enlarged posterior fossa and polydactyly detected by prenatal US. Further fetal brain MRI presented more severe anomalies, resulting in the couple deciding to terminate the pregnancies. This shows the significance of fetal brain MRI in prognosis evaluation.

Since the embryologic development of the cerebellum extends over a long time, it is more vulnerable to a wide range of destructions and disruptions (Andescavage et al., 2017). Undoubtedly, sonography is the initial imaging tool for screening of the fetal brain. However, prenatal sonography has some difficulties in subdividing multiple similar abnormalities in the cerebellum and posterior fossa due to technical limitations (Wald et al., 2004). Fetal brain MRI, which has higher-contrast resolution than prenatal sonography, has been an adjunct to prenatal ultrasound since the 1980s (Thickman et al., 1984). The use of fetal MRI has increased the diagnostic accuracy of fetal brain abnormalities (Wilson et al., 2020). Griffiths et al. (2017) demonstrated that in the United Kingdom, the overall diagnostic accuracy for detecting an isolated posterior fossa abnormality was only 65.4% for US compared to 87.7% for fetal MRI, and the prognosis was altered after MRI in 44% of cases. In 2021, another large single-center study found that fetal MRI resulted in a change in fetal prognosis in 70% of cases (Schlatterer et al., 2021). In conclusion, it is necessary to use MRI as a secondary technique to confirm, correct, or complement questionable US findings in cases of fetal brain abnormalities.

In our study, CMA revealed no clinically relevant deletions or duplications in the proband (II-2). The fetus (II-3) revealed a 696 kb deletion of 5p15.33 Supplementary Figure S3, which was inherited from his mother and identified as a VUS according to the CNV classification guidelines of ACMG. Although the 696 kb deletion encompasses four genes, none of those genes is related to fetal phenotypes. In addition, the mother did not exhibit the disease phenotypes corresponding to these genes, further indicating that these genes do not seem to be dose-sensitive and that normal expression of an allele can maintain normal biological function. To further explore the pathogenic factors, trio-WES was adopted for the proband (II-2) and the parents. A novel hemizygous non-sense variant c.2848A>T (p.Lys950Ter) of the *OFD1* gene was detected in the proband (II-2), and his mother carried this variant. Sanger sequencing confirmed that this variant was in genotype-phenotype segregation among the family members. To our knowledge, this mutation has not been reported in any public databases. *OFD1* (NM_003611.3) c.2848A>T (p.Lys950Ter), which occurred in the 21st exon, is predicted to encode a non-sense mutation leading to a premature stop codon, p.Lys950Ter. The conservation of the affected residue was relatively maintained across mammal species (Figure 3A).

To test whether this mutation caused non-sense-mediated mRNA decay and thereby loss of *OFD1* function, we measured *OFD1* mRNA levels by RNA-seq in the mother (I-2), who was a female carrier. Since the *OFD1* gene escapes X inactivation in humans according to previous reports (de Conciliis et al., 1998; Carrel et al., 1999; Carrel and Willard 2005), X inactivation should not be skewed in carrier females. Indeed, in carrier female I-2 and female control samples, there was no preferential expression of either X chromosome. Second, after calculating the statistics of reads with specific SNP sites in the control female sample, the proportion of escaped allele *OFD1* expression exceeded more than half of the normal active *OFD1* allele expression. This implied that this escape was partial and incomplete. Third, the results also showed that *OFD1* gene expression and the ratio of mutant reads to wild-type reads in the mother sample were lower than those in female control samples. These results indicated that the mutant transcripts were partially degraded. Suggesting that this truncation mutation could trigger the NMD pathway. The NMD mechanism may be one of the reasons for the reduction in the proportion of mutant transcripts. As a truncation mutation, it may result in the synthesis of abnormal protein, which can be toxic to cells through dominant negative effects. To our knowledge, carriers I-2 and II-4 in this family are both without obvious disease-associated phenotypes, and we can conclude that this abnormal protein would not cause a dominant negative effect and would only retain residual wild-type activity. Alternatively, even if it can cause this effect, the NMD mechanism would reduce the amount of abnormal protein, resulting in a loss-of-function effect (Khajavi et al., 2006).

Moreover, as predicted by AlphaFold software, the non-sense variant may lead to a change in the *OFD1* protein structure (Figure 3F). It is tempting to speculate that the function of the protein may be affected. The OFD1 gene encodes a 1011-amino acid protein that does not have similarities to previously known proteins. There is a Lis1 homology (LisH) motif in the N-terminal region and five coiled-coil α-helical domains among almost the entire length of the molecule (de Conciliis et al., 1998). As reported, the C-terminal OFD1 region containing the last two predicted coiled-coil motifs (aa 615-1012) was found to bind to the C-terminal region of SDCCAG8 (aa 533-713), which also carried two predicted coiled-coil motifs (Otto et al., 2010). At the protein level, which is predicted by AlphaFold, the substitution (Lys950Ter) would give rise to a truncated protein missing an alpha helix compared to the wild-type protein. Since the coiled-coil motifs are built by two or more alpha-helices that wind around each other to form a supercoil (UniProtKB), the lack of an alpha-helix may influence the formation of coiled-coil motifs and affect the binding of the OFD1 protein to other proteins. Thus, if a male fetus inherits this variant allele, the amount of *OFD1* mRNA will be reduced, and the protein produced by the remaining mRNA will be altered.

Mutations in OFD1 can cause four diseases, OFD1 syndrome, JBS10, RP23, and PCD. OFD1 syndrome is caused by dominant mutations of OFD1, and the last three diseases are caused by recessive OFD1 mutations. RP23 is limited to ocular symptoms, with no extraocular manifestations (Wang et al., 2017; Chen et al., 2018). PCD is a motile ciliopathy that mainly causes respiratory phenotypes (Bukowy-Bieryllo et al., 2019). OFD1 syndrome and JBS10 are primary cilia dysfunction diseases that affect multiple organs. They have both overlapping and unique phenotypes. The overlapping phenotypes presented in JBS10 and OFD1 syndrome still have some notable differences. As Gangaram et al. (2022) reported, the degree of ID is more severe and the structural CNS malformations are more extensive in JBS10 than in OFD1 syndrome. CNS malformations reported in JBS10, which are summarized in Table 2, include cerebellar vermis hypoplasia (with or without MTS), Dandy-Walker malformations, ventriculomegaly, cephalocele, meningocele, brain stem hypoplasia, polymicrogyria, microgyria, hypothalamic hamartoma, corpus callosum hypoplasia, and pituitary hypoplasia. Corpus callosum anomalies are the only commonly reported CNS malformations in OFD1 syndrome. Polydactyly is often seen in JBS10 but is uncommon in OFD1 syndrome, while polycystic kidney is much more prevalent in OFD1 syndrome.

**TABLE 2:** Prenatal and postnatal features of individuals with previously described OFD1 variants in JBS10 (including the individuals in this study).

**No.**	Gender	Race	Age	Prenatal phenotypes			Postnatal phenotypes		PMID	Exon intron	Nucleotide change	Type of mutation	Predicted protein
				Prenatal ultrasound	Brain MRI	Autopsy	Brain MRI	Clinical manifestation					
1	M	NA	NA	NA			MTS, encephalocele	Intellectual impairment, hypotonia,ataxia, polydactyly	26092869 (Juric-Sekhar et al., 2012)	Exon 3	c.149A>G	Missense	p.H50R
2	M	Caucasian	22 week	Cephalocele, congenital hepatic fibrosis, cleft soft palate,post-axial polydactyly	No MTS vermis hypoplasia enlarged posterior fossa small occipital meningocele	Brain: Dorsal enlargement of medulla oblongata, pons flat, punctuate periventricular hemorrhage; Left recent intraventricular hemorrhage; Kidney: Slightly enlarged and tortuous ureters bilaterally, renal minimal tubular dilation and no cyst formation; Liver: enlarged tortuous bile ductules in the portal tracts with fibrosis	TP: termination of pregnancy		22331178 (Juric-Sekhar et al., 2012)	Exon 3	c.277G>T	Missense	p.V93F
3	M	Caucasian	20.6 week	Cephalocele congenital hepatic fibrosis	No MTS, vermis hypoplasia enlarged posterior fossa, small occipital meningocele	Brain: dorsal enlargement of medulla oblongata, hypoplastic basal ganglia; Kidney: Slightly enlarged and tortuous ureters bilaterally, renal minimal tubular dilation and no cyst formation; Liver: enlarged tortuous bile ductules in the portal tracts with fibrosis	TP: termination of pregnancy		22331178 (Juric-Sekhar et al., 2012)	Exon 3	c.277G>T	Missense	p.V93F
4	M	African American	Prenatal: 22 week Postnatal: 10 day	Macrocephaly severe lateral ventriculomegaly posterior fossa malformation micrognathia suspicion of a cleft palate	MTS meningocele abnormal configuration of the brainstem superior cerebellar peduncles cervical	Absence of the pituitary gland severe adrenal hypoplasia	MTS severe ventriculomegaly, brainstem hypoplasia Dandy-Walker malformation, bilateral polymicrogyria empty sella no normal pituitary gland tissue	Respiratory failure Facial region: macrocephalic, frontal bossing apparent hypertelorism,low-set and plastic ears, Pierre Robin Sequence(micrognathia, midline cleft palate, glossoptosis); Abdomen region: small omphalocele small microphallus; ophthalmological examination: hypertelorism microphthalmia with microcornea, coloboma; renal ultrasound: bilateral mild hydronephrosis, no adrenal glands; endocrine evaluation: panhypopituitarism	30895720 (Aljeaid et al., 2019)	Exon 6	c.515 T>C	Missense	p.L172P
5	M	Japanese	NA	NA			MTS	Hypotonia pure JS	27434533 (Suzuki et al., 2016)	Exon 7	c.537_539del	Inframe del	p.D181del
6	M	Chinese	24 week	NA	MTS enlarged cystic fourth ventricle enlarged lateral ventricles(hydrocephalus)	NA	TP: termination of pregnancy		32944789 (Zhang et al., 2021)	Exon 7	c.599 T>C	Missense	p.L200P
7	M	Chinese	24 week	Tetralogy of Fallot	MTS	NA	TP: termination of pregnancy		32944789 (Zhang et al., 2021)	Exon 7	c.599 T>C	Missense	p.L200P
8	M	Chinese	4 year	NA	NA	NA	MTS macrogyria	apnea and feeding difficulties in infancy severe motor developmental delay intellectual disability	32944789 (Zhang et al., 2021)	Exon 7	c.599 T>C	Missense	p.L200P
9	M	NA	NA	MCA (multiple congenital abnormality)	MTS	NA	TP: termination of pregnancy		26275793 (Westerfield et al., 2015)	Exon 7	c.604_609delGAGTAT	Inframe del	p.E202_Y203del
10	M	Australian	Prenatal: second trimester Postnatal: 7 year	Macrocephaly with non-progressive ventriculomegaly	NA	NA	MTS hydrocephalus	Kidney: polycystic kidney disease at 5y, awaiting transplantation; ocular region: hypermetropia, intermittent esotropia dysarthria, dyspraxia mild intellectual impairment relatively well-preserved non-verbal cognitive abilities.	22353940 (Field et al., 2012)	Exon8	c.688-705del18	Inframe del	p.230-235del IKMEAK
11	M	Australian	11 year	NA			MTS polymicrogyria	Macrocephalic asymmetric spasticity in his upper limbs significant dysarthria and dyspraxia borderline/low non-verbal cognitive skills afebrile focal dyscognitive seizure at 4.5y increased echogenicity of kidneys at 6.5y	22353940 (Field et al., 2012)	Exon8	c.688-705del18	Inframe del	p.230-235del IKMEAK
12	M	French Canadians	15 year	NA	NA	NA	MTS	Oculomotor apraxia, renal Involvement hypotonia, ataxia	26477546 (Srour et al., 2015)	Exon9	c.920 T>A	Missense	p.V307D
13	M	Hispanic descent	17 year	NA			MTS small cerebellum vermis Dandy Walker malformation with enlarged posterior fossa, marked ventriculomegaly absence of the corpus callosum	Intellectual disability seizure disorder, complex polydactyly renal failure small optic nerves with colobomas facial anomalies profound sensorineural hearing loss tongue hamartoma repaired cleft palate	27081566 (Wentzensen et al., 2016)	Exon11	c.1129 + 4A>T	Frameshift	p.T353Kfs*13/p.K354Nfs*4
14	Not mention								30581852 (Linpeng et al., 2018)	Exon17	c.2321-2322insT	Frameshift	p.S790P*X802
15	M		9 year	Hydronephrosis (spontaneously resolved by 2 years)	Polymicrogyria cerebellar vermis hypoplasia	NA	MTS hypothalamic hamartoma(HH) small pituitary gland partial thinning corpus callosum diffuse polymicrogyria	Neonatal feeding difficulties undescended testes developmental delay growth failure hypotonia facial anomalies polydactyly pneumonia and recurrent lung infections isolated growth hormone deficiency Ocular region: bilateral colobomas	35398350 (Gangaram et al., 2022)	Exon18	c.2484dupT	Non-sense	p.Glu829*
16	M	Chinese	22 week	Hypoplastic cerebellum absent vermis with enlarged lateral ventricles postaxial polydactyly	NA	NA	TP: termination of pregnancy		30581852 (Linpeng et al., 2018)	In18	c.2488 + 27>C	Splicing	
17	Not mention								30581852 (Linpeng et al., 2018)	In19	c.2582dupT	Splicing	
18	M	Japanese	NA	NA			MTS	Moderate intellectual disability Severe developmental delay retinal dystrophy and coloboma	27434533 (Suzuki et al., 2016)	Exon20	c.2629-2632del	Frameshift	p.E878Kfs*9
19	not mention								30581852 (Linpeng et al., 2018)	Exon20	c.2632-2635delGAAG	Frameshift	p.E878del
20	M	American	5 year	NA			MTS meningocele malformation of brainstem structures	Macrocephaly; facial region**:** low-set ears, Oculomotor apraxia, Epicanthal folds, telecanthus neonatal breathing dysregulation, central apnea, delayed psychomotor development intellectual disability hypotonia Self-mutilation Submucosal cleft palate Notched upper lip or tongue Oral motor dysfunction rockerbottom feet hypertricosis moderate hearing loss gastroesophageal reflux Hip dysplasia	28344780 (Kane et al., 2017)	Exon20	c.2656delC	Frameshift	p.G886Kfs*2
21	M	Malaysian	12 year	NA			MTS occipital encephalocele corpus callosum shortening	Macrocephaly tongue thrusting distinctive craniofacial features polydactyly, relative brachydactyly, fetal finger pads, small feet newborn feeding difficulties severe intellectual disability ambulation difficulties hyperphagia with obesity lateral deviation and contractures at the knees mild peripheral hypotonia reduced tendon jerks mild dysmetria optic nerve atrophy pulmonary infections	19800048 (Coene et al., 2009)	Exon20	c.2767delG	Frameshift	p.E923Kfs
22	M		13 year	NA			MTS	mental retardation behavioral troubles(anxiety and intolerance to frustration) oculomotor apraxia polydactyly brachydactyly obesity chronic sinusitis and bronchitis(recurrent infections)	23036093 (Thauvin-Robinet et al., 2013)	Exon21	c.2789-2793del	Frameshift	p.I930Kfs*8
23	M	NA	34 week	Macrocephaly postaxial polydactyly thoracic situs inversus cerebellar vermis hypoplasia hypothalamic hamartoma (HH) polysplenia	NA	NA	TP:termination of pregnancy		23036093 (Thauvin-Robinet et al., 2013)	Exon21	c.2797dupG	Frameshift	p.E933Gfs*7
24	M	Malaysian	NA	NA			MTS	Central apnea, feeding problems Facial region: low-set ears, facial anomalies(a broad nasal bridge, prominent philtrum and maxillary arch, full lips) mental retardation postaxial polydactyly juvenile retinitis pigmentosa conductive hearing impairment	19800048 (Coene et al., 2009)	Exon21	c.2841_2847del	Frameshift	p.K948NfsX8
25	M	Chinese	3M21D				MTS	Six finger deformity, short limbs, coloboma of optic disc and choroid situs inversus	28173652 (Meng et al., 2017)	Exon21	c.2843-2844delAA	Frameshift	p.K948RfsX
26	M								19800048 (Coene et al., 2009)	Exon21	c.2844-2850del	Frameshift	p.K948Nfs*9
27	M	Chinese	30 week	Thickening of the superior cerebellar feet cerebellar vermis agenesis enlarged posterior fossa polydactyly	MTS meningocele thin brain stem polymicrogyria	NA	NA		This article	Exon21	c.2848A>T	Non-sense	p.Lys950Ter
28	M	Chinese	25 week	Cerebellar vermis agenesis enlarged posterior fossa polydactyly	MTS meningocele thin brain stem polymicrogyria	Cerebellar vermis agenesis polydactyly	NA		This article	Exon21	c.2848A>T	Non-sense	p.Lys950Ter

**FIGURE 4:**
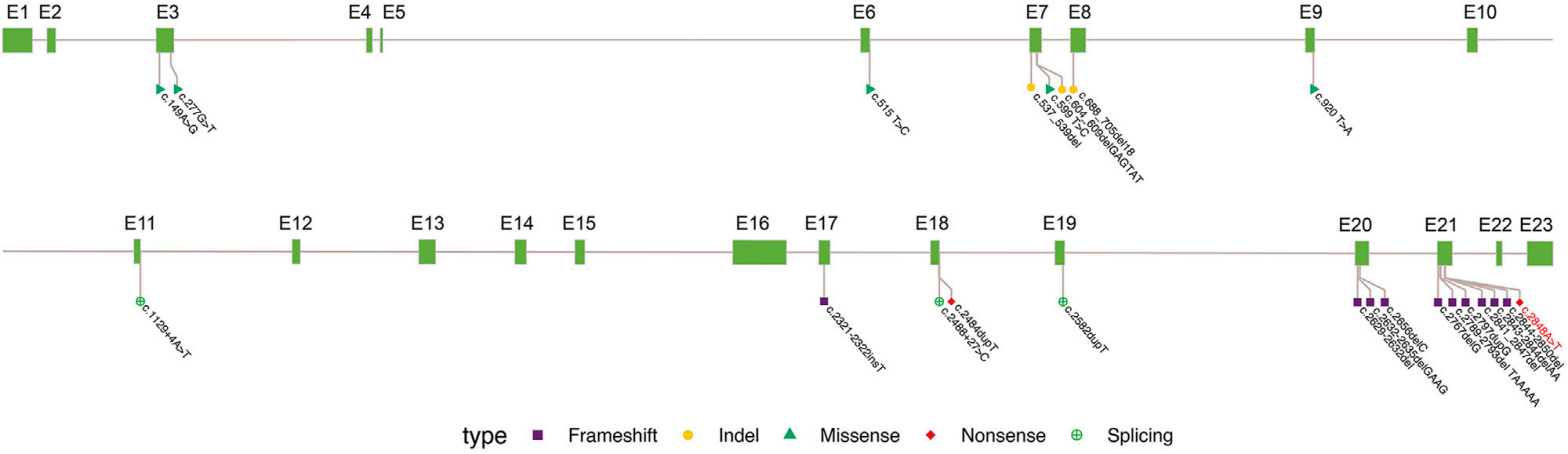
Summary of OFD1 variations in JBS10. Exon (grass–green square); variation types: frameshift (purple square), indel not causing frameshift (yellow circle), missense (green triangle), non-sense (red diamond), and synonymous (green cross circle). The site marked in red is reported in the paper.

To date, only 23 variants of *OFD1* have been reported to cause JBS10 (Figure 4), including 10 deletion/insertion mutations, four in-frame deletions, five missense mutations, two splicing mutations and two non-sense mutations. Mutations, especially frameshift mutations in the first 16 exons of the *OFD1* gene, mostly lead to OFD1 syndrome. OFD1 syndrome is an X-linked dominant disease that shows typical dysmorphic features, malformations of the oral cavity, and skeletal and CNS abnormalities in female patients (Franco and Thauvin-Robinet 2016; Pezzella et al., 2022). However, it is often lethal in males. Notably, the five missense mutations (*OFD1* c.149A>G, c.277G>T, c.515 T>C, c.599 T>C, c.920 T>A) and the three in-frame deletions (*OFD1* c.537_539del, c.604_609del, c.688-705del) shown in Table 2, which were located in the first 16 exons but caused JBS10, not *OFD1* syndrome, may have occurred because these mild mutations may not lead to *OFD1* protein truncation and likely do not abolish its binding affinity to functionally interacting proteins, such as lebercilin (Coene et al., 2009; Bachmann-Gagescu et al., 2015; Kroes et al., 2016). Other mutations are located up to exon 17 of the protein, mainly in exon 21, generating proteins retaining the fifth coiled-coil domain and predicting reduced binding of the protein to its partners without affecting pericentriolar localization (Coene et al., 2009). In addition, as shown in Table 2, there is a frameshift mutation (*OFD1* c.1129 + 4A>T) in exon 11 of Patient 13 presenting as JBS10 rather than *OFD1* syndrome. Another frameshift mutation (*OFD1* c.2789-2793del) in exon 21 presented as JBS10 in one patient but PCD in another. The mechanism accounting for this phenomenon requires further study.

The phenotypic spectrum associated with *OFD1* mutations resulting in JBS10 has been extended over the years. To date, 28 patients from 23 families with JBS10 have been identified (Table 2), four of whom had no phenotypic description in the articles (Patients 14, 17, 19, and 26). The fetal phenotypes were obtained in 16 fetuses, and there were mainly brain abnormalities, such as MTS, meningocele, polymicrogyria, thin brainstem, ventriculomegaly, and macrocephaly. Some fetuses (31%, 5/16) had polydactyly, and other anomalies were also found in the individual fetuses. Overall, 12% (2/16) of fetuses had congenital hepatic fibrosis; 6% (1/16) had tetralogy of Fallot; 6% (1/16) presented with hypothalamic hamartoma (HH), thoracic situs inversus and polysplenia; 6% (1/16) had cleft palate; and 6% (1/16) displayed hydronephrosis. Postnatal phenotypes were obtained in 15 male patients. Common clinical manifestations among these patients included facial anomalies, intellectual impairment, motor developmental delay, hypotonia, ataxia, seizure, polydactyly, renal involvement, retinal dystrophy and colobomas, hearing impairment, cleft palate, pulmonary infections, dyspnea and newborn feeding difficulties. Panhypopituitarism was seen in Patient 4, and HH was present in Patient 15. When we encounter a fetus with the dominant prenatal phenotypes described above, we should consider the possibility of genetic disease, and chromosome detection alone is not sufficient. Trio-WES should be performed on these fetuses to identify etiological factors in genetics. In addition, when gestation was less than 22 weeks (Patients 2 and 3), the MTS was not obvious, and we should be vigilant about the suspected brain signs. When a fetus has been diagnosed with JBS10, possible postnatal phenotypes should be provided to the couple to evaluate prognosis.

## Conclusion

In this study, we first identified a novel variant of the *OFD1* gene employing WES technology and bioinformatics strategies, broadening the spectrum of *OFD1* mutations. Our results provide data for genetic counseling and prenatal diagnosis in this family and contribute to comprehending the possible pathogenic mechanisms of JBS10, which is beneficial to genetic counseling and timely perinatal management.

## Data Availability

The data presented in the study are deposited in the NCBI BioProject repository, accession number PRJNA914088.

## Supplementary material

SUPPLEMENTARY FIGURE S1 Assessment of the pathogenicity of variation OFD1 (NM_003611.3): c.2848A>T (p.Lys950Ter) by VarSome (https://varsome.com/).

SUPPLEMENTARY FIGURE S2 The predicted structure of the mutant protein and wild-type protein by RoseTTAFold online software. **(A)** (wild-type protein), **(B)** (mutant protein).

SUPPLEMENTARY FIGURE S3 The CMA analysis of the fetus (II-3), which shows a 696 kb heterozygous deletion at 5p13.33 (2091630-2787829).
