# Supplementary material for: A novel non-sense variant in the OFD1 gene caused Joubert syndrome
Source: Front Genet. 2023 Jan 10;13:1064762. doi: 10.3389/fgene.2022.1064762 (PMC9871390; doi:10.3389/fgene.2022.1064762)
Supplement: Supplementary file 1 [file DataSheet1.zip › 12-21Supplementary Figures and table/Supplementary Table S1.docx]

Table S1. Evaluation of the pathogenicity of the novel variant c.2848 A>T in the *OFD1* gene.

|  | OFD1 c.2848A>T (p.Lys950Ter) |
| --- | --- |
| Population data | Absent in population GnomAD (v2.1.1) databases (PM2_Supporting) |
| Computational and predictive data | Nonsense, predicted to undergo NMD (PVS1) |
| Functional data | NA |
| Segregation data | NA |
| De novo data | NA |
| Allelic data | NA |
| Other databases | Not reported in Mastermind or LitVar |
| Other data | NA |
| Conclusion | Likely Pathogenic (PVS1 + PM2_Supporting) |

NA, not available
