# Supplementary figures and images for: A novel non-sense variant in the OFD1 gene caused Joubert syndrome
Source: Front Genet. 2023 Jan 10;13:1064762. doi: 10.3389/fgene.2022.1064762 (PMC9871390; doi:10.3389/fgene.2022.1064762)

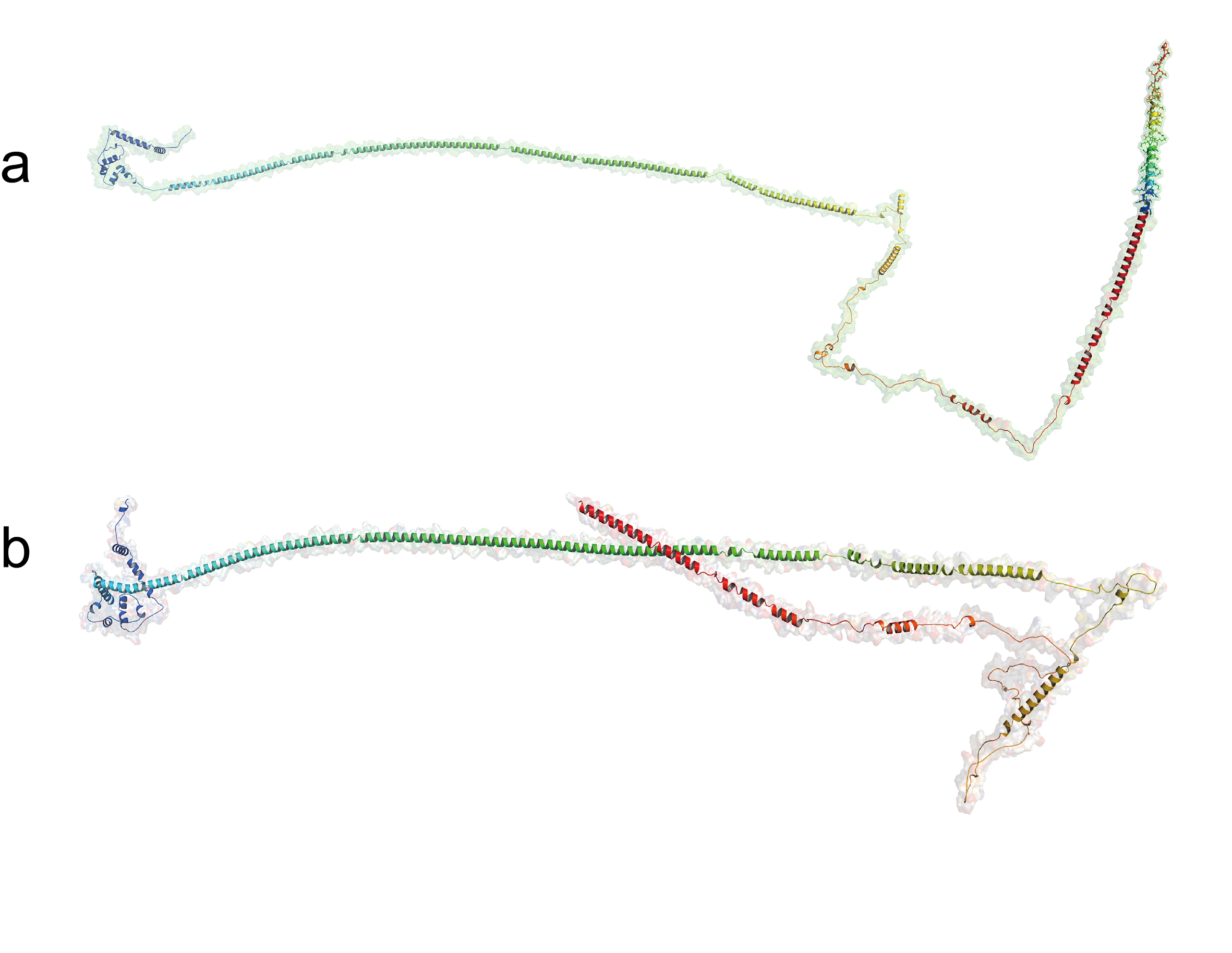

Supplement: Supplementary file 1 [file DataSheet1.zip › 12-21Supplementary Figures and table/Supplementary Figure S1.jpg]

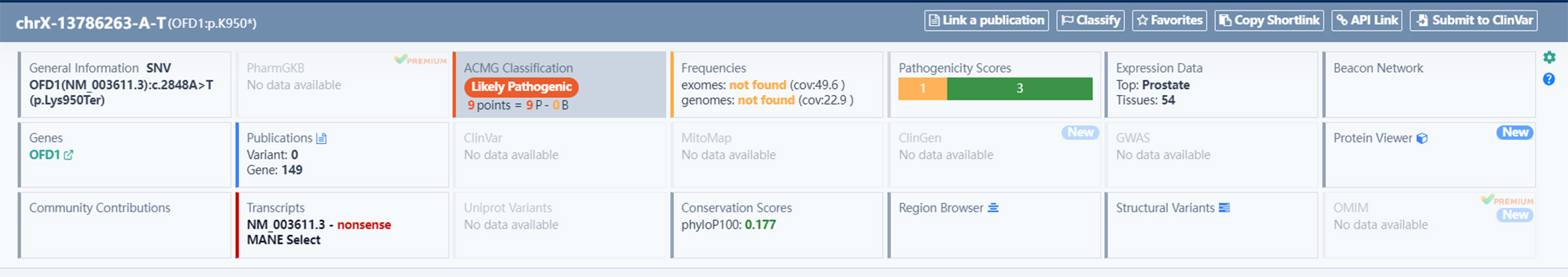

Supplement: Supplementary file 1 [file DataSheet1.zip › 12-21Supplementary Figures and table/Supplementary Figure S2.jpg]

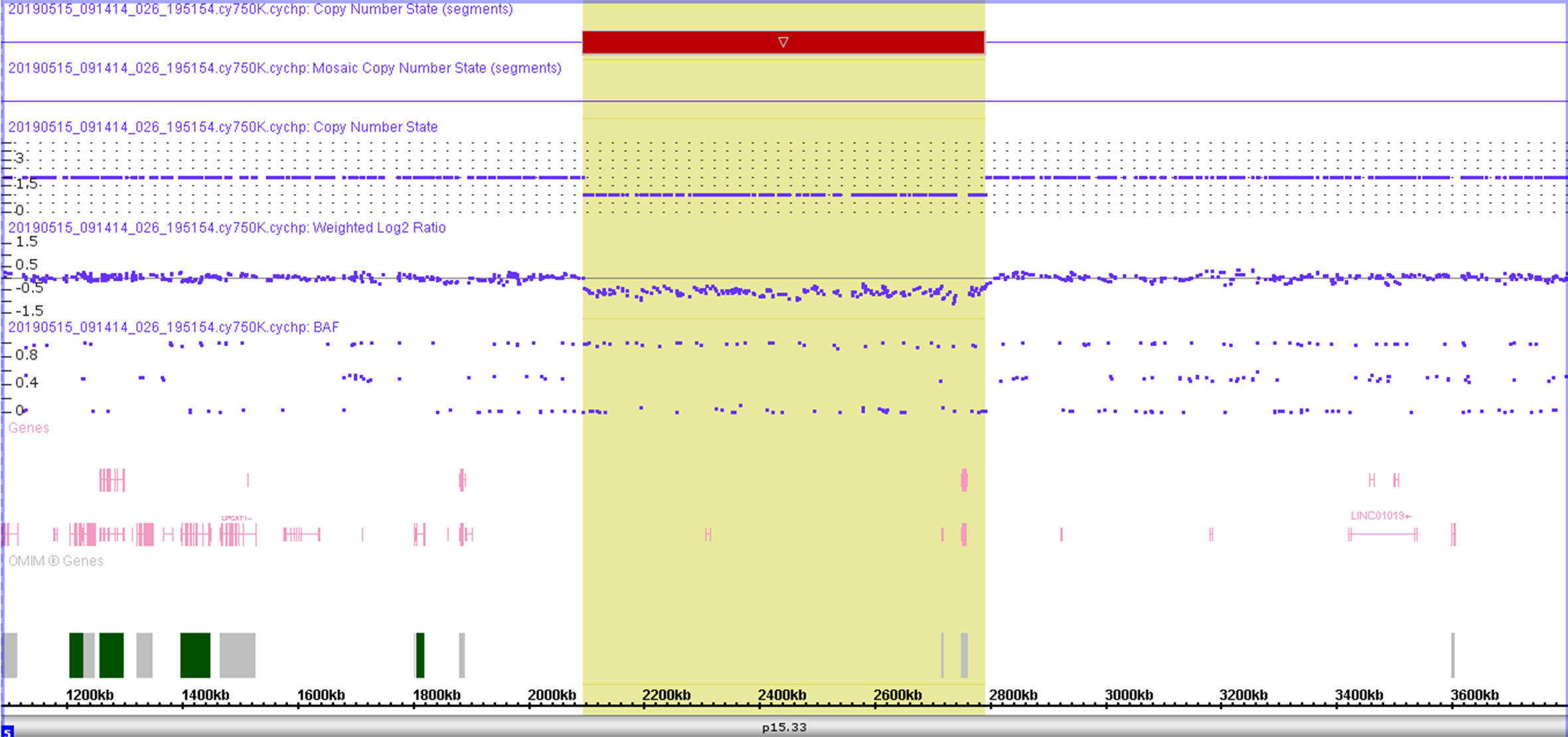

Supplement: Supplementary file 1 [file DataSheet1.zip › 12-21Supplementary Figures and table/Supplementary Figure S3.jpg]
